# RNA sequence and length contribute to RNA-induced conformational change of TLS/FUS

**DOI:** 10.1038/s41598-020-59496-0

**Published:** 2020-02-14

**Authors:** Nesreen Hamad, Tsukasa Mashima, Yudai Yamaoki, Keiko Kondo, Ryoma Yoneda, Takanori Oyoshi, Riki Kurokawa, Takashi Nagata, Masato Katahira

**Affiliations:** 1 Institute of Advanced Energy, Kyoto University Kyoto, 611-0011 Japan; 2 Graduate School of Energy Science, Kyoto University Kyoto, 606-8501 Japan; 3 Research Center of Genomic Medicine, Saitama Medical University, Saitama, 350-0495 Japan; 4 Department of Chemistry, Graduate School of Science, Shizuoka University, 836 Ohya, Suruga, Shizuoka, 422–8529 Japan

**Keywords:** DNA-binding proteins, RNA-binding proteins, Structural biology

## Abstract

Translocated in liposarcoma (TLS)/fused in sarcoma (FUS) is a multitasking DNA/RNA binding protein implicated in cancer and neurodegenerative diseases. Upon DNA damage, TLS is recruited to the upstream region of the cyclin D1 gene (*CCND1*) through binding to the promotor associated non-coding RNA (pncRNA) that is transcribed from and tethered at the upstream region. Binding to pncRNA is hypothesized to cause the conformational change of TLS that enables its inhibitive interaction with histone acetyltransferases and resultant repression of *CCND1* expression, although no experimental proof has been obtained. Here, the closed-to-open conformational change of TLS on binding pncRNA was implied by fluorescence resonance energy transfer. A small fragment (31 nucleotides) of the full-length pncRNA (602 nucleotides) was shown to be sufficient for the conformational change of TLS. Dissection of pncRNA identified the G-rich RNA sequence that is critical for the conformational change. The length of RNA was also revealed to be critical for the conformational change. Furthermore, it was demonstrated that the conformational change of TLS is caused by another target DNA and RNA, telomeric DNA and telomeric repeat-containing RNA. The conformational change of TLS on binding target RNA/DNA is suggested to be essential for biological functions.

## Introduction

Translocated in liposarcoma (TLS), also known as fused in sarcoma (FUS), belongs to the TET or FET family (TLS/FUS, Ewing’s sarcoma, and TATA-binding protein-associated factor), whose members are multitasking RNA/DNA binding proteins that regulate gene expression, and are implicated in cancer and neurodegenerative diseases. TLS was first identified in a human adipose tissue tumor, a myxoid liposarcoma, as a fusion oncogenic protein that results from chromosomal translocation t(12;16)(q13.3;p11.2)^1^. Later, TLS was found to be involved in various devastating neurodegenerative diseases^2–6^, as well as in a wide variety of biological processes through regulation of gene expression^7–15^.

TLS reportedly binds to a wide variety of RNA and DNA sequences, both structured and unstructured, that are involved in transcription, splicing, and so on^9^. Previously, systematic evolution of ligands through exponential enrichment (SELEX) showed that TLS recognizes the GGUG motif^16^, while a photoactivatable ribonucleoside-enhanced crosslinking immunoprecipitation (PAR-CLIP) study and RNA-binding competition study identified an AU-rich stem-loop structure and CGCGC sequence, respectively, as additional targets for TLS^17,18^. More recently, normalization of CLIP-seq (CLIP combined with deep sequencing) data by means of Nascent-seq data showed a combination of six motifs (UGUG, CUGG, UGGU, GCUG, GUGG, and UUGG) are also targeted by TLS^19^.

**Figure 1:**
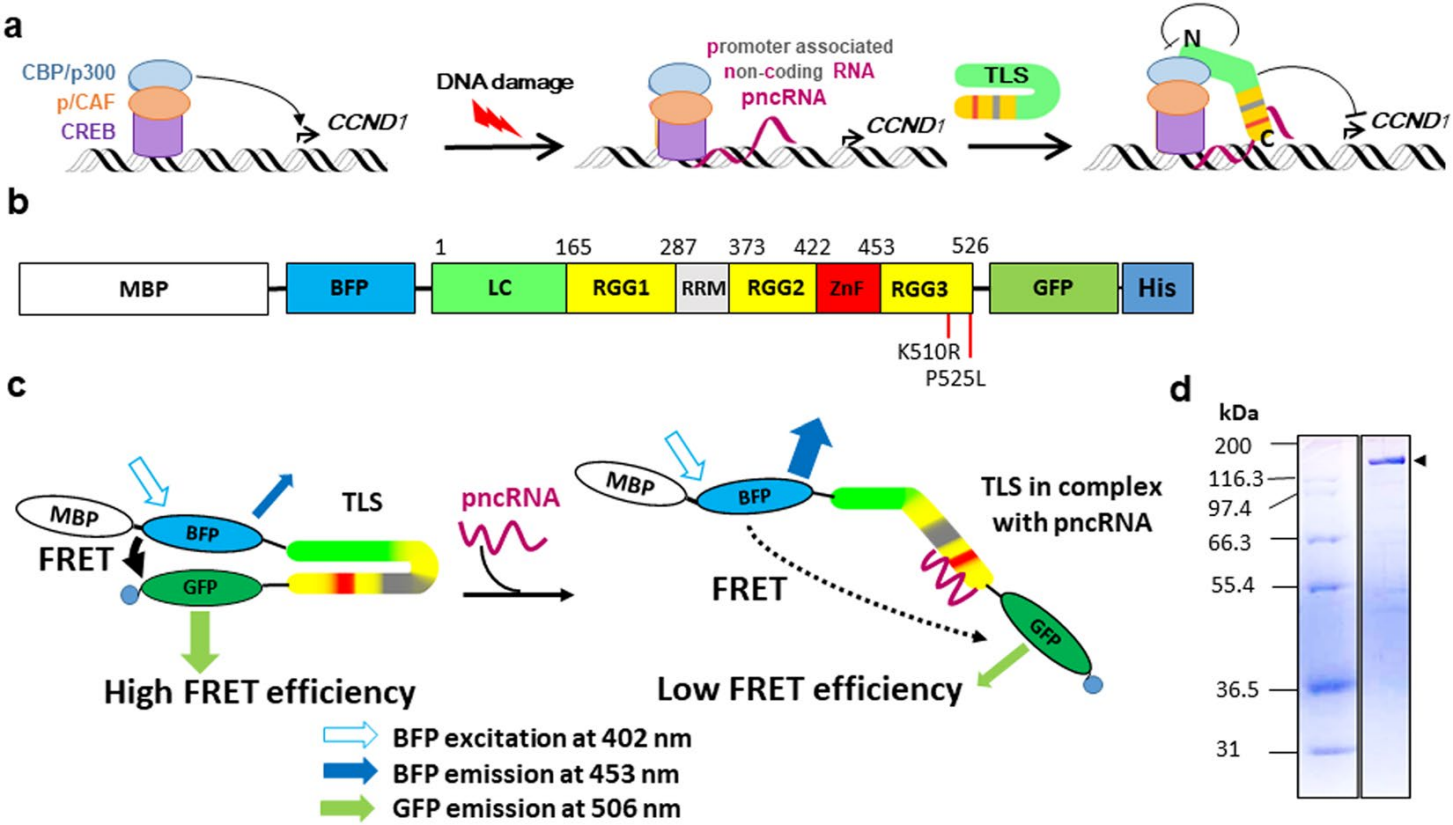
The hypothesized closed-to-open conformational change of TLS on binding to pncRNA for the repression of *CCND1* and the strategy for the detection of the conformational change by FRET. (**a**), The mechanism of the repression of *CCND1* by TLS on DNA damage^7^. A closed-to-open conformational change of TLS is hypothesized on binding pncRNA, which enables TLS to interact with CBP/p300 and repress their histone acetyltransferase activities, resulting in the repression of *CCND1*. (**b**), Schematic illustration of the protein used in this study, MBP-BFP-TLS-GFP-6xHis. Residue numbers of TLS are indicated. Positions of ALS-linked mutations, K510R and P525L, are also indicated. (**c**), The strategy for the detection of the closed-to-open conformational change of TLS by FRET. (**d**), 10% SDS-PAGE of MBP-BFP-TLS-GFP-6xHis, arrowhead (right), and the molecular mass markers (left). These are cropped gels. The uncropped full-length gel is presented in Supplementary Fig. S2 online.

TLS comprises from the N- to C- terminal ends, a low complexity domain (LC), the first Arginine-Glycine-Glycine rich motif (RGG1), an RNA recognition motif (RRM), the second RGG (RGG2), a Zinc finger domain (ZnF), and the third RGG (RGG3) (Fig. 1b). The LC and RGGs are known to be unstructured and therefore are called disordered regions. Additionally, RGG, RRM, and ZnF are well known nucleic acid binding domains, and the corresponding domains of TLS were recently shown to play a central role in RNA and DNA binding. Loughlin *et al*. recently determined the structures of ZnF and RRM, each in a complex with RNA^20^. They showed that ZnF recognizes the GGU sequence, while RRM is more shape-specific than sequence-specific, RRM showing highly degenerate sequence specificity for a NYNY quartet (N = any nucleotide, Y = C or U). It was also shown by isothermal titration calorimetry (ITC) that RRM does not exclusively bind to stem-loop structures, but also binds to single-stranded sequences, like AU-rich or GC repeats, as mentioned above. Additionally, the RGG2 portion of RRM-RGG2 was found to interact with the 5′ single-stranded loop region of the stem-loop structure and to disrupt the sequential base-base stacking, thereby destabilizing the RNA structure^21^. NMR studies thus have provided a structural basis for RNA recognition by individual nucleic acid-binding domains of TLS.

TLS is reportedly involved in shortening of the telomere, which is located at the end of the human chromosome, through repression of telomerase-independent telomere-elongation^12^. In this biological process, TLS was found to be included in the ternary complex with telomeric DNA and telomeric repeat-containing RNA (TERRA), whose sequences are d(TTAGGG)n and r(UUAGGG)n, respectively. This ternary complex recruits histone trimethyltransferases Suv3–9 h and Suv4–20 h, by which lysine 20 of H4 (H4K20) and lysine 9 of H3 (H3K9) are trimethylated, resulting in promotion of heterochromatinization of the telomere, subsequent inhibition of homologous recombination of telomeric DNA, and telomere shortening^12,19,22–24^. It was shown previously that RGG3 of TLS is responsible for the formation of the ternary complex; Tyr residues and Phe residues of RGG3 are involved in the specific recognition of TERRA and telomeric DNA, respectively^24^. Our NMR-based binding assay revealed the interactions in the binary and ternary complexes of RGG3 with telomeric DNA or/and TERRA, which suggested that the plastic roles of tyrosine and phenylalanine are important for RGG3 to efficiently form the ternary complex^25^. It now seems important to investigate the relative roles of the different RNA binding domains and also other regions of TLS in the context of the full-length protein to understand the molecular mechanism by which TLS regulates various different biological processes.

**Figure 2:**
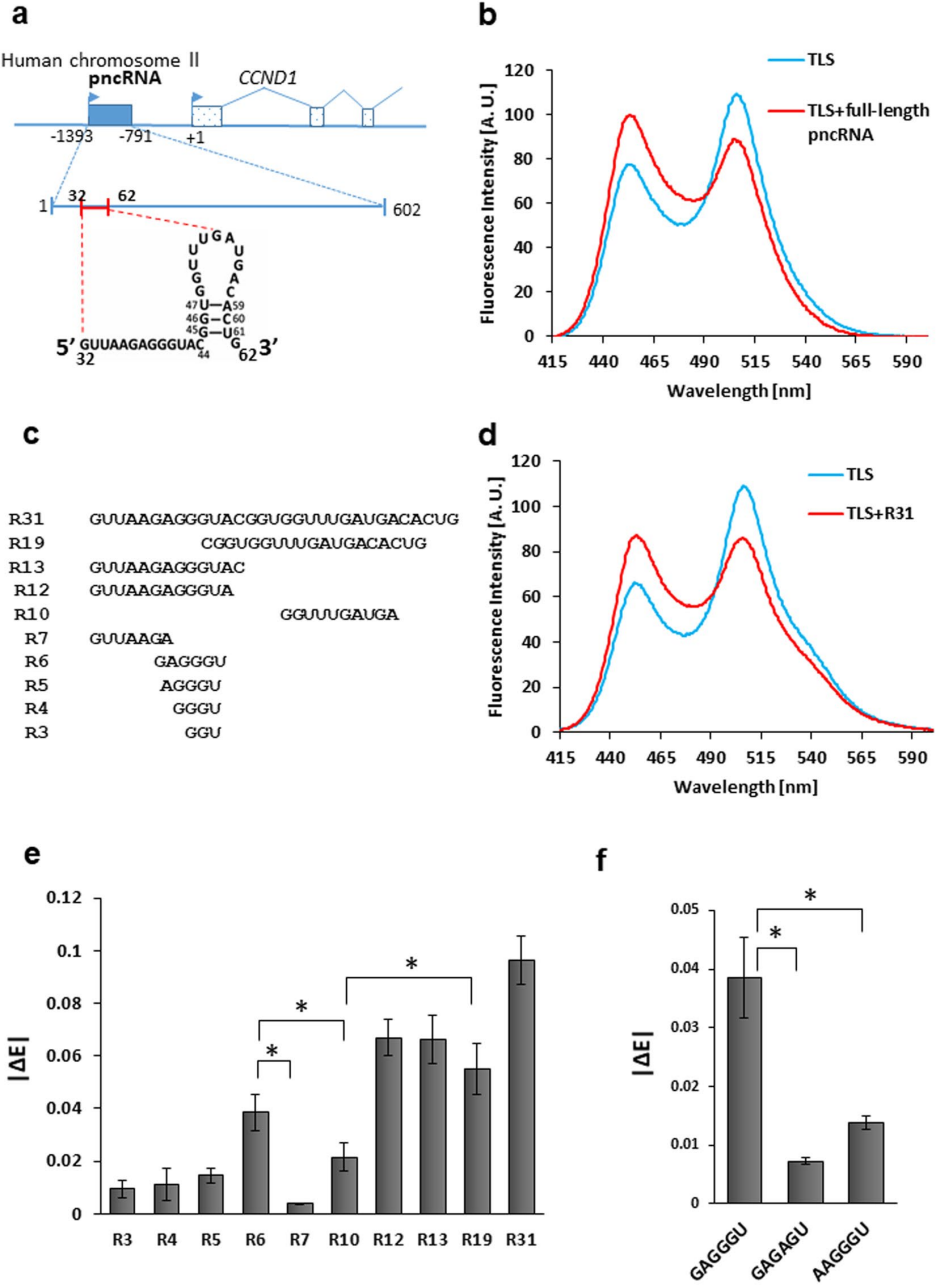
Detection of the closed-to-open conformational change of TLS on binding of pncRNA by FRET. (**a**), The localization, length, sequence and secondary structure of pncRNA^21^. (**b**), Fluorescence spectrum of TLS (100 nM), excited at 402 nm, in either the absence (blue) or presence (red) of an equimolar amount of full-length pncRNA of 602 nt. (**c**), The names and sequences of fragments of R31 of pncRNA. (**d**), Fluorescence spectrum of TLS in either the absence (blue) or presence (red) of an equimolar amount of R31 of pncRNA. (**e**), Absolute values of the change in the relative FRET efficiency, ∆*E*, where *E* = *I*_GFP_/ (*I*_GFP_ + *I*_BFP_), on the addition of each fragment of R31 of pncRNA. Three independent results were averaged for each fragment. |∆*E*| is presented as a mean ± standard deviation. *indicates *p* value < 0.05. (**f**), |∆ *E*| of R6, GAGGGU, and its mutants.

Previously, Wang *et al*. reported that TLS downregulates the transcription of *CCND1* in response to DNA damage^7^, which is supposed to result in prevention of DNA replication and cell-cycle progression. DNA damage triggers the transcription of long non-coding RNA (lncRNA) of 602 nucleotides from the 5′ upstream region of *CCND1*, named pncRNA (Fig. 1a). Subsequently, TLS is recruited to the 5′ upstream region of *CCND1* through binding to pncRNA. Binding of pncRNA also relieves the N-terminal region of TLS from masking by the C-terminal region of TLS, allowing the N-terminal region of TLS to bind to CREB-binding protein (CBP)/E1A-binding protein P300 (p300) and to repress the histone acetyltransferase HAT activity of CBP/p300 allosterically. Accordingly, Wang *et al*. suggested a model where pncRNA serves as a ligand for TLS, causing an allosteric effect to release it from an inactive conformation, which in turn allows gene-specific TLS–CBP/p300 interactions resulting in inhibition of the HAT activity of CBP/p300 and the repression of *CCND1* transcription. Later, we found that a portion of pncRNA (nucleotide numbers 32–62) comprising 31 nucleotides, named pncRNA D1–1, is sufficient for binding of TLS, and demonstrated that the 5′ half of pncRNA D1–1 takes on a single-stranded structure and that the 3′ half forms a stem-loop structure (Fig. 2a)^21^. Here, we hypothesize that TLS undergoes structural changes, i.e. from a “closed” to an “open” conformation, upon binding to pncRNA (Fig. 1a) and pncRNA D1–1, and investigate this hypothesis experimentally.

To investigate the structural changes of TLS upon addition of various nucleic acids, we performed fluorescence resonance energy transfer (FRET) assay. FRET is a methodology that can determine the distance between two chromophores, a donor and acceptor. If the distance between the donor-acceptor pair is less than 10 nm, FRET efficiency is high, whereas FRET efficiency is poor if the distance exceeds more than 10 nm^26,27^. The FRET assay is useful to obtain information on the average of all possible conformations at a certain time. FRET assay has previously been applied to unfolded and intrinsically disordered proteins to characterize the ensembles of disordered states and their transitions to partially or fully ordered states^28,29^. To undertake the FRET assay, we introduced blue fluorescent protein (BFP) and green fluorescent protein (GFP) to the N- and C-termini of TLS, respectively (Fig. 1b). Additionally, maltose binding protein (MBP) was added to the N-terminus of BFP to enhance the solubility of TLS and a 6xHis-tag was attached to the C-terminus of GFP for purification. We expect that TLS takes on a closed form when free, in which GFP and BFP are supposed to be in close proximity, and shows high FRET efficiency (Fig. 1c, left). On the other hand, upon binding with the target RNA, we expect that TLS takes on an open form, in which GFP and BFP are supposed to be far apart, and shows low FRET efficiency (Fig. 1c, right).

In this study, we firstly demonstrated by means of the FRET assay that TLS really undergoes the conformational change, extension, on binding to pncRNA. The extension is implied to correspond to the closed-to-open conformational change of TLS. We then identified a critical region of pncRNA for induction of the conformational change of TLS. We also investigated the effect of the length of RNA on conformational change of TLS. Subsequently, we investigated the effects of other important TLS targets, telomeric DNA and TERRA, both of which form the G-quadruplex structure, on the conformational change of TLS. Finally, we examined whether ALS-linked mutations have any effects on either the conformation or conformational change of TLS.

## Results

### Implication of the closed-to-open conformational change of TLS on binding to pncRNA

MBP-BFP-TLS-GFP-6xHis protein was successfully expressed in E. coli cells and purified by Ni-affinity chromatography followed by size-exclusion chromatography (SEC). Monomer fractions of the protein in SEC were collected referencing the elution volume of the molecular marker standard (see Supplementary Fig. S1). Figure 1d and Supplementary Fig. S2 online show the result of SDS-PAGE of the purified protein. Hereafter, MBP-BFP-TLS-GFP-6xHis protein is simply called TLS.

Firstly, the conformational change of TLS caused by binding of the full-length pncRNA (602 nucleotides) was examined by FRET. Figure 2b shows the fluorescence spectrum of TLS excited at 402 nm. In addition to the peak of BFP at around 453 nm, a peak of GFP at around 506 nm was observed due to FRET (Fig. 2b). On the addition of the full-length pncRNA, the fluorescence intensity at 506 nm decreased, while that at 453 nm increased (Fig. 2b). This reflects the reduction in FRET, which indicates the increase in the BFP-GFP distance. The increase in the BFP-GFP distance means some kind of extension of TLS. It is implied that TLS undergoes the closed-to-open conformational change on binding to pncRNA, which enables TLS to interact with CBP/p300 for the repression of expression of the *CCND1* gene.

Secondly, the conformational change of TLS caused by binding of pncRNA D1–1 (31 nucleotides, Fig. 2a), R31 (Fig. 2c), was examined by FRET. The spectral change of TLS caused by R31 turned out to be very similar to that by the full-length pncRNA (Fig. 2d). This indicates that R31 is sufficient to cause the same extent of conformational change of TLS as the full-length pncRNA. Thus, it was revealed that R31 is sufficient not only for binding of TLS but also for causing the conformational change of TLS.

The remaining protein after the FRET experiments was re-injected to SEC, which ensured that protein exists in the monomer state over the experimental time. This indicates that the observed FRET is intramolecular one, not intermolecular one.

MBP is fused to increase the solubility. No RNA binding activity was observed for MBP protein^23,30^. The effect of MBP on the FRET results was examined. MBP was cleaved by thrombin and the resultant protein was purified by SEC. The fluorescence spectrum and its change on the addition of R31 were very similar for TLS with and without MBP (see Supplementary Fig. S3). These results indicated that the presence of MBP does not affect the FRET results of TLS.

### Identification of a critical region of pncRNA for induction of the conformational change of TLS

In order to identify a critical region of pncRNA for induction of the conformational change of TLS, various fragments of R31 (Fig. 2c and Supplementary Table S1) were tested. The relative FRET efficiency, also known as proximity ratio, *E* = *I*_GFP_ / (*I*_GFP_ + *I*_BFP_), was calculated in either the presence or absence of each fragment, and the change in *E* caused by each RNA fragment was obtained. |Δ*E*| can be regarded as a measure to estimate the extent of the conformational change of TLS caused by RNA. It should be noted that R6 caused a larger conformational change of TLS than either R7 or R10, although R6 is shorter (Fig. 2e). It is supposed that the GAGGGU region of pncRNA is critical for induction of the conformational change of TLS. When a single mutation was introduced at R6, reduction in |Δ*E*| was observed for GAGAGU and AAGGGU (mutations are underlined) (Fig. 2f). This indicated that R6 causes a conformational change of TLS in a sequence-specific manner.

It is remarkable that |Δ*E*| of R19 is much larger than that of R10. R19 can form the stem-loop structure shown in Fig. 2a, while R10 cannot due to the lack of the residues that form the stem structure. It is suggested that the stem-loop structure in the 3′ region of R31 is also important for the induction of the conformational change of TLS.

It was seen that R12 caused a larger conformational change of TLS than R19, although the difference was not statistically significant (Fig. 2e). As R12 is shorter than R19, the larger or at least comparable |Δ*E*| value observed for R12 may indicate that the 5′ single-stranded region of R31 has slightly more pronounced effect on the conformational change of TLS than the 3′ stem-loop region.

### The DNA counterpart of pncRNA causes less conformational change of TLS

**Figure 3:**
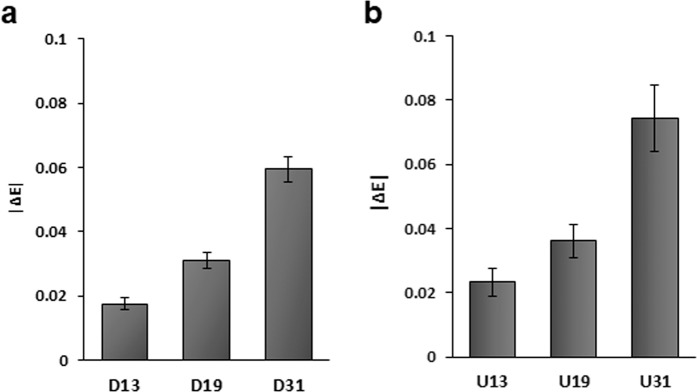
The conformational change of TLS caused by either DNA counterparts of the fragments of pncRNA or oligomers of U residues of various lengths. (**a**), |∆*E*| of DNA counterparts, D13, D19 and D31. Three independent results were averaged for each fragment. |∆*E*| is presented as a mean ± standard deviation. (**b**), |∆*E*| of oligomers of U residues of various lengths, U_13_, U_19_ and U_31_.

D13 is a DNA counterpart of R13 of pncRNA (see Supplementary Table S1). |Δ*E*| of D13, 0.0175 ± 0.0018 (Fig. 3a), is rather smaller than that of R13, 0.0663 ± 0.0091 (Fig. 2e). Similarly, |ΔE| of D19, 0.0309 ± 0.0025 (Fig. 3a), is rather smaller than that of R19, 0.0550 ± 0.0099 (Fig. 2e), and |ΔE| of D31, 0.0594 ± 0.0039 (Fig. 3a), is rather smaller than that of R31, 0.0964 ± 0.0092 (Fig. 2e). The conformational change of TLS on binding of the DNA counterpart of pncRNA turned out to be much less.

### The effect of the length of RNA on the conformational change of TLS

It was reported that the affinity of TLS to RNA is affected not only by the presence of a specific sequence but also by the length of non-specific RNAs^23^. Therefore, we examined the effect of the length of non-specific RNAs on the conformational change of TLS. It was revealed from the results in Fig. 2f that G residues play a specific role in the conformational change of TLS on binding. Thus, oligomers of U residues were chosen as representative non-specific RNAs. It was found that the longer the non-specific RNA is, the larger the |ΔE| value is (Fig. 3b). That is, a long non-specific RNA causes a larger conformational change of TLS than a short non-specific RNA.

R13 is a part of pncRNA (R31) and thus is regarded as a specific sequence (Fig. 2c). U_13_ is a non-specific version of R13 of the same length. |ΔE| of U_13_ (0.0233 ± 0.0043) is smaller than that of R13 (0.0663 ± 0.0091) (Figs. 2e and 3b). The R13/U_13_ ratio of |ΔE| is 2.8. Similarly, R19 and U19 are regarded as specific and non-specific sequences of the same length. |ΔE| of U_19_ (0.0361 ± 0.0051) is smaller than that of R19 (0.0550 ± 0.0099) (Figs. 2e and 3b), the ratio being 1.5. R31 and U_31_ are also regarded as specific and non-specific sequences of the same length. |ΔE| of U_31_ (0.0743 ± 0.0103) is smaller than that of R31 (0.0964 ± 0.0092) (Figs. 2e and 3b), the ratio being 1.3. It should be noted that the specific/non-specific ratio of |ΔE| becomes smaller when the length of RNA becomes greater. That is, the difference in the extent of the conformational change of TLS caused by RNA between specific and non-specific sequences becomes smaller when the length of RNA increases.

### Comparison of the affinity of TLS toward specific and non-specific RNAs

**Figure 4:**
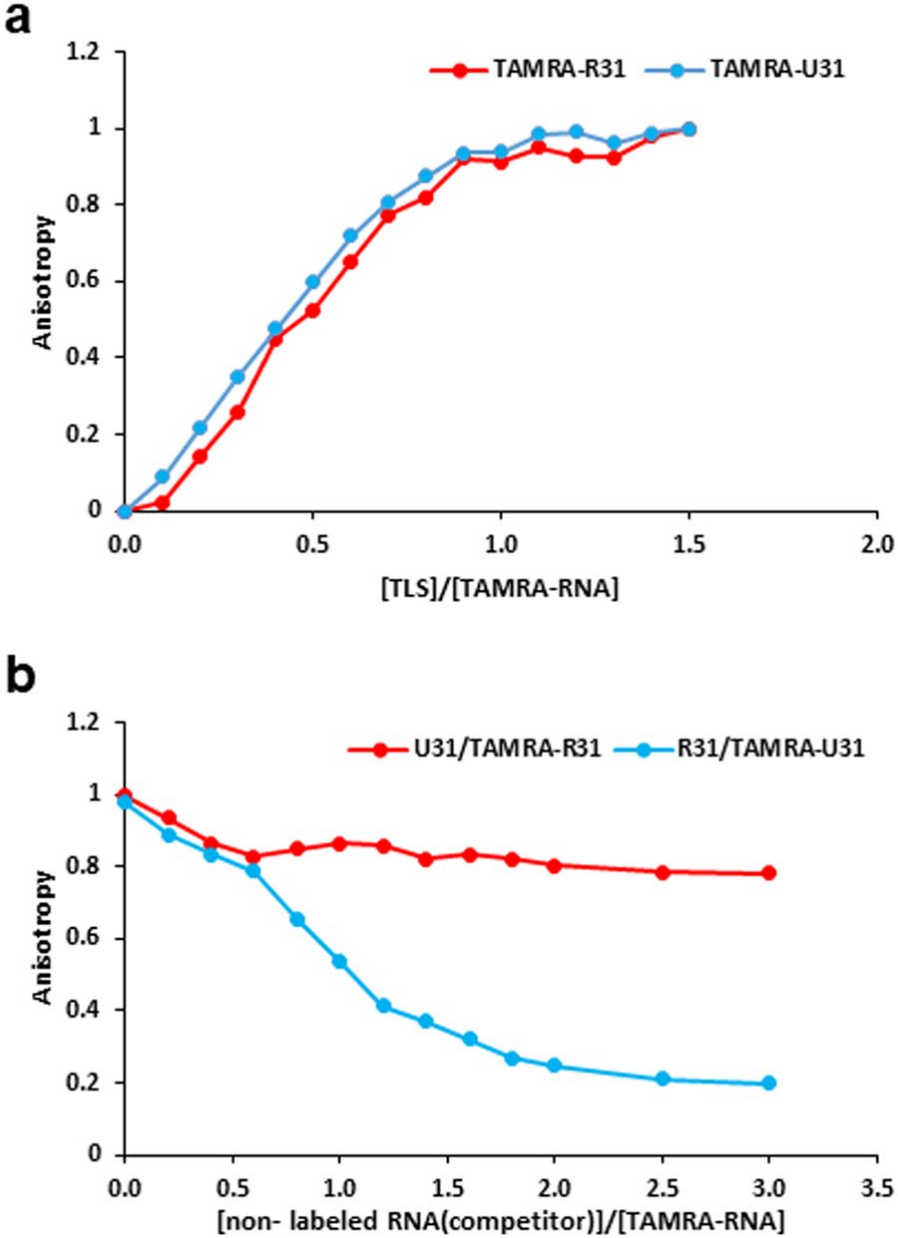
The difference in affinity toward TLS between a specific RNA, R31 of pncRNA, and non-specific RNA, U31. (**a**), Fluorescence anisotropy of TAMRA-labeled R31 (100 nM) (red) and TAMRA-labeled U31 (100 nM) (blue) in the course of the addition of TLS. (**b**), Fluorescence anisotropy of TAMRA-labeled R31 (100 nM) (red) in the presence of a 1.5 molar ratio of TLS in the course of the addition of non-labeled U31 and that of TAMRA-labeled U31 (100 nM) (blue) in the presence of a 1.5 molar ratio of TLS in the course of the addition of non-labeled R31.

The affinity of TLS toward specific R31 and non-specific U_31_ was compared. Firstly, the affinity was compared through the titration of TLS against either TAMRA-labeled R31 or TAMRA-labeled U_31_ using fluorescence anisotropy of the complex. First of all, it should be noted that on the basis of the excitation/emission spectra of both BFP^31^ and GFP^32^, the light of 546 nm used for the excitation of TAMRA does not result in excitation of either BFP or GFP. Due to the complex formation between TLS and either R31 or U_31_ during the titration, the fluorescence anisotropy increased (Fig. 4a). The difference in affinity between R31 and U_31_ was not evident under the experimental conditions applied.

Then, the difference in affinity was examined by means of competition experiments. When R31 was added to the solution containing the TLS-U_31_ complex, the anisotropy largely decreased (blue line in Fig. 4b). This indicates that U_31_ that was bound to TLS was mostly replaced by the added R31. When U_31_ was added to the solution containing the TLS-R31 complex, on the other hand, the anisotropy decreased just a little (red line in Fig. 4b). This indicates that R31 that was bound to TLS was not replaced by the added U_31_ but that was still mostly bound to TLS. These competition results demonstrated that the affinity of TLS toward specific R31 is higher than that toward non-specific U_31_.

### The effect of the G-quadruplex structure of DNA/RNA on the conformational change of TLS

**Figure 5:**
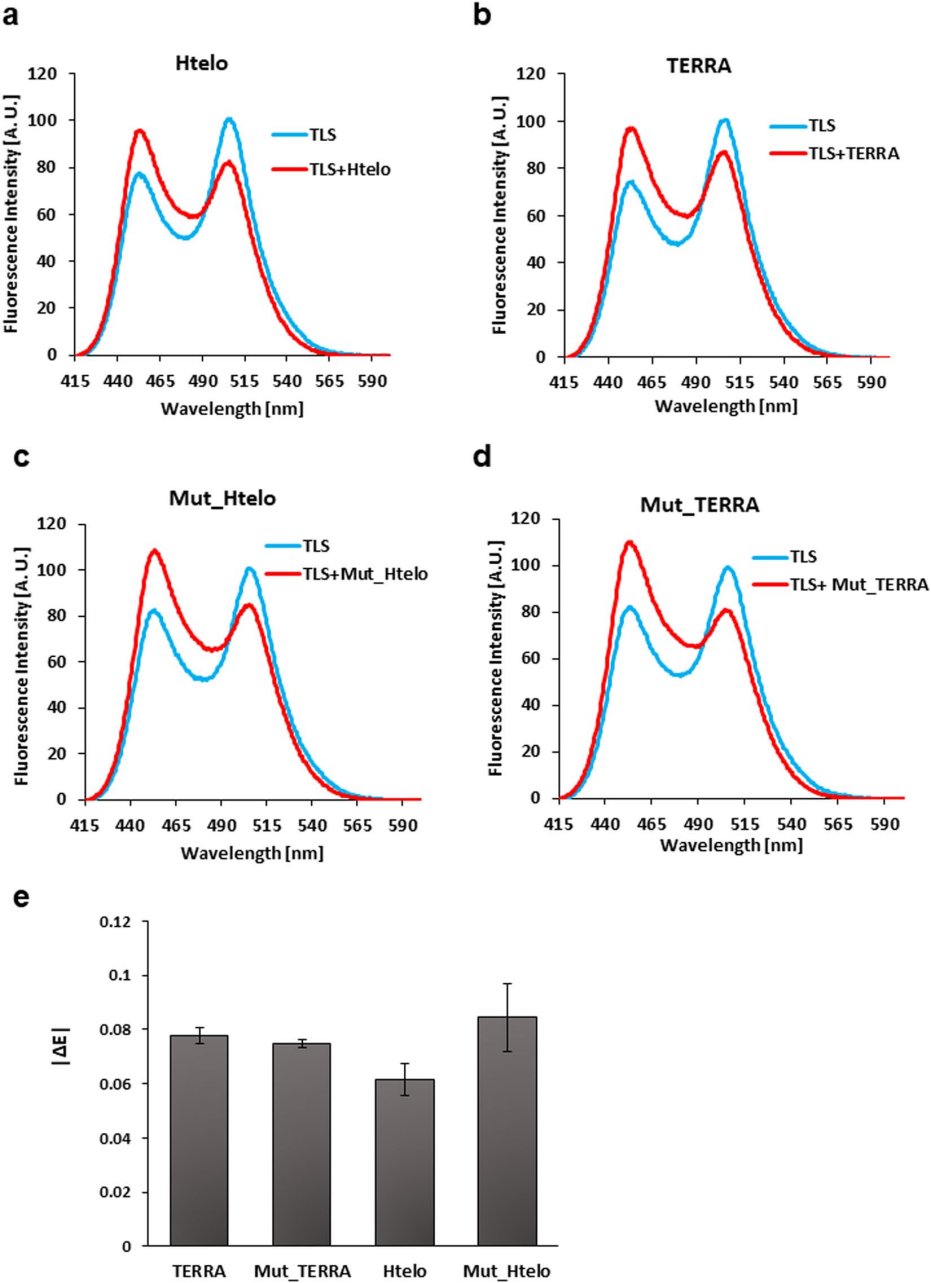
The conformational change of TLS caused by TERRA and telomeric DNA. Fluorescence spectrum of TLS in either the absence (blue) or presence (red) of TERRA (**a**), telomeric DNA (**b**), mutant TERRA (**c**), or mutant telomeric DNA (**d**). (**e**), |∆*E*| of each RNA or DNA.

It was reported that TLS interacts with either telomeric DNA or TERRA, both of which form the G-quadruplex structure^12,22,25^. Therefore, the conformational changes of TLS caused by these DNA and RNA were examined. The sequences of TERRA and telomeric DNA (Htelo) are shown in Supplementary Table S1 online. It was revealed that both TERRA and telomeric DNA cause the implicated closed-to-open conformational change of TLS (Fig. 5a,b), |ΔE| being 0.0776 ± 0.0029 and 0.0614 ± 0.0059, respectively (Fig. 5e).

Then, the necessity of the presence of the G-quadruplex structure for the conformational change of TLS caused by TERRA and telomeric DNA was examined. Two G residues of TERRA were replaced by U residues to produce a mutant TERRA, Mut_TERRA (Supplementary Table S1). Similarly, two G residues of telomeric DNA were replaced by T residues to produce a mutant telomeric DNA, Mut_Htelo (Supplementary Table S1). It has already been revealed that both mutants do not form the G-quadruplex^33^. It was found that both mutant TERRA and telomeric DNA can cause a similar conformational change of TLS (Fig. 5c,d), |ΔE| being 0.0748 ± 0.0013 and 0.0845 ± 0.0124, respectively (Fig. 5e). These results indicate that the presence of the G-quadruplex structure is not critical for the conformational change of TLS caused by either TERRA or telomeric DNA.

### ALS-linked mutation does not affect either the conformation or the conformational change of TLS

TLS mutations are found in ~5% of familial ALS patients^5^. The effect of the mutations on either the conformation of TLS in the absence of RNA or the extent of the conformational change induced by pncRNA was examined. Two mutants reported in ALS patients, P525L and K510R^34,35^, were tested. The positions of the mutations are shown in Fig. 1b. The mutant proteins were successfully expressed and purified in the same way as the wild-type protein. The fluorescence spectrum of either P525L or K510R was similar to that of the wild-type. This indicated that both the mutants take on a structure that is similar to that of the wild-type protein. |ΔE| of either P525L or K510R on binding of R31 was also close to that of the wild-type (see Supplementary Fig. S4). Thus, it was found that ALS-linked mutations do not affect either the conformation of TLS or the extent of the conformational change induced by pncRNA.

## Discussion

In order to explain the biological function of TLS, the closed-to-open conformational change of TLS on binding of pncRNA was suggested (Fig. 1a)^7^, although no experimental proof was available. Here, FRET results indicated for the first time that TLS actually undergoes a conformational change, extension, on binding of the full-length pncRNA of 602 nucleotides (Fig. 2b). It is implied that this extension corresponds to the closed-to-open conformational change.

We assume that the driving force for the formation of the closed conformation is the electrostatic interactions between the LC domain and RGG domains. The isoelectric point (pI) of the LC domain estimated by the ExPASy program^36^ is 3.56, indicating that the LC domain is negatively charged at neutral pH. On the other hand, pI values of RGG1, RGG2 and RGG3 are 9.30, 12.13 and 11.02, respectively, indicating that all RGG domains are positively charged under neutral pH. Thus, electrostatic interactions between the LC domain and RGG domains are likely. The Π-cation interaction between tyrosine residues in the LC domain and arginine residues in the RGG domains^36^ may also contribute to the formation of the closed conformation. As RNA is negatively charged, we assume that pncRNA binds to the positively charged RGG domains and disrupts the interactions between the LC domain and RGG domains, resulting in the disruption of the closed conformation and the transformation to the open conformation.

We revealed that the RRM domain does not interact with pncRNA, while the RGG3 domain does^21^. The interaction of the ZnF domain with a certain RNA was reported, in which involvement of N435, F438 and R441 was suggested^20^. Therefore, the effect of the mutation for these residues was examined. The |Δ*E*| value of the mutant in which all of the three residues, N435, F438 and R441, and a M436 residue were mutated to alanine residues was 0.0991, which turned out to be almost comparable to the |Δ*E*| value of the wild-type for R31. This may support the importance of the interactions involving RGG domains with pncRNA for the conformational transformation, which is described above.

It would be added that the extension of TLS in which the N-terminal region of TLS is relieved from masking by the C-terminal region of TLS can account for the experimental transcriptional repression, even if the view of the closed-to-open conformational change of TLS may be oversimplified. It would be also added that the conformational change may not be a simple two-state transition but that the FRET results may indicate that the population of the extended conformations of TLS increased on binding of the pncRNA.

It is remarkable to find that a small portion of pncRNA, pncRNA D1–1 of 31 nucleotides (R31), is sufficient to cause this complete conformational change of TLS (Fig. 2d), although it has already been shown that R31 is sufficient for binding of TLS^21^.

The dissection of R31 revealed that the GAGGU sequence located in the 5′ half of R31 is critical for the conformational change of TLS (Fig. 2e). In particular, the importance of G residues for the conformational change of TLS was revealed by mutagenesis (Fig. 2f). The fact that the sequences recognized by TLS are mostly rich in G residues^16,18–22^ may be biologically relevant in the context of the importance of G residues for causing the conformational change of TLS. The stem-loop structure in the 3′ half of R31 was also identified to be critical for the conformational change of TLS (Fig. 2a,e).

It was revealed that the DNA counterpart of pncRNA causes less conformational change of TLS than pncRNA (Fig. 3a), which indicates that the difference in a sugar type affects the conformational change of TLS. This may be related to the previous report that RNAs bind to TLS more strongly than DNA counterparts^23^.

It was found that the extent of the conformational change of TLS caused by RNA is governed not only by a specific sequence but also by the length of a non-specific sequence. Long oligo(U) RNA causes a larger conformational change of TLS than shorter oligo(U) RNA (Fig. 3b). Apparently, in addition to the presence of the specific RNA sequence, the length of non-specific RNA is another factor that determines the extent of the conformational change of TLS. This finding may be related to the previous report that regardless of the presence of a certain sequence or structure, the length of RNA/DNA dominates the affinity toward TLS in some cases^23^.

When the RNA is short, the extent of the caused conformational change of TLS largely differs between specific and non-specific sequences. When the RNA is long, on the other hand, the extent of the caused conformational change of TLS does not differ so much between specific and non-specific sequences (Figs. 2e and 3b). This can be interpreted by that for the conformational change of TLS, the RNA sequence factor is dominant when the RNA is short, while the RNA length factor is dominant when the RNA is long. Thus, the extent of the conformational change of TLS is supposed to be governed by two factors, sequence and length.

In order to address the biological significance of specific R31 over non-specific U_31_, the affinity toward TLS was compared between R31 and U_31_. The competition experiment indicated that specific R31 exhibits higher affinity toward TLS than non-specific U_31_ (Fig. 4b). Therefore, it is assumed in cells that a specific RNA such as R31 can interact with TLS due to high affinity, while a non-specific RNA such as U_31_ cannot due to low affinity. Then, as a matter of fact, the conformational change of TLS can be caused only by a specific RNA, but not by a non-specific RNA. Thus, practically, only a specific RNA may exert the biological function.

There could be two models regarding the relationship between binding of RNA and the RNA-induced conformational change. In the first model, the binding and the conformational change are concerted. In the second model, the binding is not sufficient to trigger the conformational change. All the data presented can be basically explained by the first model. Nonetheless, we do not completely abandon the contribution of the second model in a certain case, in order to consider more biological significance of specific RNA.

Induction of the conformational change of TLS was examined not only for pncRNA but also for other targets of TLS, TERRA and telomeric DNA, that are known to form the G-quadruplex structure. It was found that TERRA and telomeric DNA cause a similar extent of conformational change of TLS as pncRNA (Fig. 5a,b,d). It is suggested that the conformational change of TLS might be generally caused by target DNA/RNA and that the conformational change may be essential for TLS to exert certain biological functions through the target DNA/RNA.

It was also found that the formation of the G-quadruplex structure is not needed for either TERRA or telomeric DNA to cause the conformational change of TLS (Fig. 5c,d,e). It was reported that TLS binds to the G-quadruplex structure more strongly than the single-stranded structure^22,24^, although this is controversial^23^. Therefore, it may be still a case that the conformational change of TLS caused by either TERRA or telomeric DNA having the G-quadruplex structure is biologically significant, if only the interaction with high affinity may be able to occur in cells.

The possible correlation between the occurrence of ALS and the change in either the conformation of TLS or the extent of the conformational change of TLS caused by RNA was examined. FRET studies did not show any correlation between them.

## Materials and Methods

### Preparation of oligonucleotides

The sequences of the oligonucleotides used in FRET and fluorescence anisotropy analyses are shown in Supplementary Table S1 online. Full-length pncRNA was *in vitro* transcribed by MEGAscript Kit (AM1334, Thermo Fisher Scientific) according to manufacturer’s protocol. Briefly, pncRNA sequence was inserted to pcDNA 3.1 ( + ) vector (V790–20, Thermo Fisher Scientific), and RNA were transcribed by T7 transcription and purified by phenol/chloroform extraction, followed by isopropanol precipitation. The deletion mutants of pncRNA (TAMRA-labeled and non-labeled) and their DNA counterparts (non-labeled) were synthesized, purified, and de-salted by FASMAC Co., Ltd. TERRA, mutant TERRA, Htelo, mutant Htelo, A_12_, G_12_, U_12_, and C_12_ were obtained from Hokkaido System Science Co., Ltd. The sequences of the primers used for subcloning are shown in Supplementary Table S2 online. The primers were synthesized and purified by Invitrogen.

### Plasmid construction

The DNA sequence coding for the TLS was inserted between the genes encoding the maltose binding protein (MBP) and the hexahistidine tag (6xHis) of the pMAL-p2x vector. Then the genes encoding blue fluorescent protein (TagBFP) and green fluorescent protein (TagGFP2) were inserted between the genes encoding MBP and TLS, and between the genes encoding TLS and 6xHis, respectively. The DNA sequences coding for the TagBFP and TagGFP2 were obtained from the pCasper3-BG plasmid (Clontech) by means of polymerase chain reaction (PCR) using the primers indicated in Supplementary Table S2 online. The obtained DNA sequence encoding MBP-BFP-TLS-GFP-6xHis was confirmed by DNA sequencing. The ALS-linked TLS mutants, K510R TLS and P525L TLS, were constructed by following the QuickChange Site-directed Mutagenesis Kit protocol (Agilent Technologies) using the primers shown in Supplementary Table S2 online and the above obtained plasmid containing the MBP-BFP-TLS-GFP-6xHis gene as a template.

### Protein expression

The MBP-BFP-TLS-GFP-6xHis protein (TLS) and the two mutants, TLS K510R and TLS P525L, were expressed using BL21 (DE3) Gold Escherichia coli cells. A single colony was inoculated into 10 mL LB medium containing 100 mg/L ampicillin and grown overnight at 37 °C. The overnight culture was transferred to 1 L LB medium containing the same antibiotics and incubated at 37 °C until the OD_600_ reached ∼0.6. Then the temperature of the culture was lowered to 20 °C and expression of the protein was induced by addition of 0.1 mM isopropylthio-β-D-galactopyranoside (IPTG). The culture was incubated for an additional 20 h. Subsequently, the bacterial cells were pelleted at 1500 g and stored at −20 °C until purification.

### Protein purification

The stored cells were resuspended in lysis buffer comprising 50 mM Tris-HCl, pH 7.6, 25 mM glucose, 1% CHAPS (Wako Pure Chemical Industries, Ltd.), 10 mM benzamidine, 5 U/mL DNase I (Invitrogen), 1 mg/L RNase (Nippon Gene), and 0.2 g/L lysozyme. All the following procedures were carried out at 4 °C unless otherwise stated. Cells were lysed by sonication (Astrason ultrasonic processor, Misonix inc.) on ice. The cell lysates were centrifuged at 50 000 g for 30 min. The supernatants were purified by nickel-affinity column chromatography using Ni sepharose beads (GE Healthcare Bio-Sciences). The column was washed with wash buffer comprising 50 mM Tris-HCl, pH 7.6, 1 M NaCl, 25 mM glucose, 1% CHAPS, and 25 mM imidazole. Protein was eluted with elution buffer comprising 50 mM Tris-HCl, pH 7.6, 25 mM glucose, 1% CHAPS, and 500 mM imidazole. 10% SDS-PAGE was performed to identify the fractions containing TLS. The pooled fractions were further purified by size exclusion chromatography (SEC) using a Hiload 16/60 Superdex 200 prep grade column (GE Life Sciences). The column was equilibrated with a buffer comprising 50 mM Tris-HCl, pH 7.6, 250 mM NaCl, 25 mM glucose, and 1% CHAPS. The fractions containing monomer TLS were collected. The purity was confirmed by 10% SDS-PAGE. The protein concentration was determined from the absorbance at 280 nm using a molar extinction coefficient (ɛ) value of 185770. MBP and 6xHis tags were kept attached; MBP enhances the solubility. MBP and His tag cleavage was performed once to ensure that their presence did not affect the conformational change of TLS. The final concentration of 5 mM fresh dithiothreitol (DTT) was added to the purified TLS solution, which was then kept at 4 °C. All the experiments were carried out within one week after purification.

### Fluorescence resonance energy transfer (FRET) assay

A final concentration of 50 nM TLS dissolved in 5 mM Tris-HCl, pH 7.6, 0.1% CHAPS, 2.5 mM glucose, 25 mM NaCl, and 0.5 mM DTT was prepared in a total volume of 140 µL as a TLS sample by diluting the TLS stock solution with RNase free water. To make a sample containing 50 nM both TLS and RNA, an equimolar amount of RNA was also dissolved. Fluorescence spectra were collected with a steady-state photon counting spectrofluorometer (JASCO FP-8500 spectrometer, Japan Spectroscopic Co.) using a standard quartz cuvette with an optical path length of 1 cm. The excitation wavelength was 402 nm. The spectra slit width of 5 nm was used for excitation and emission with an integration time of 1 nm/s from 415 nm to 650 nm. All the measurements were carried out at 25 °C. A blank was measured and subtracted from all the spectra. Data were processed using a JASCO Spectra Manager for FP-8000 series. The relative FRET efficiency (*E*) for TLS, also known as proximity ratio, was calculated using the formula, *E* = *I*_GFP_/(*I*_GFP_ + *I*_BFP_), where *I*_BFP_ and *I*_GFP_ are the intensities at 453 nm for BFP and 506 nm for GFP, respectively. The effects on *I*_GFP_ of direct excitation of GFP at 402 nm and leakage of the BFP emission into the GFP emission, bleed through, were corrected following the method described in the literature^37^. The change in *E* value upon addition of RNA was calculated using the equation, ∆*E* = |*E*_+RNA_-*E*_-RNA_|, where *E*_-RNA_ and *E*_+RNA_ are the *E* values in the absence and presence of RNA, respectively.

### Fluorescence anisotropy assay

100 nM TAMRA-labeled RNA (TAMRA-RNA) in 5 mM Tris-HCl, pH 7.6, 0.1% CHAPS, 2.5 mM glucose, 25 mM NaCl, and 0.5 mM DTT was prepared in a total volume of 140 µL. Then the purified protein was titrated against TAMRA-RNA. For the competition assay, the non-labeled RNA competitor was titrated against a solution containing TLS and TAMRA-RNA, whose molar ratio was 1:1.5. Fluorescence anisotropy measurements were conducted using a FP-8500 spectrofluorometer (JASCO) with excitation at 546.0 nm and emission at 577.5 nm at 25 °C. For each titration point, fluorescence anisotropy spectra were recorded, seven times, which were automatically averaged. JASCO Spectra Manager software was used for spectral measurement. Data were exported as excel files for analyses.

## Supplementary information

Supplementary information.
