# Supplementary material for: RNA sequence and length contribute to RNA-induced conformational change of TLS/FUS
Source: Sci Rep. 2020 Feb 14;10:2629. doi: 10.1038/s41598-020-59496-0 (PMC7021683; doi:10.1038/s41598-020-59496-0)
Supplement: Supplementary file 1 — Supplementary information. [file 41598_2020_59496_MOESM1_ESM.pdf]

RNA sequence and length contribute to RNA-induced conformational change of TLS/FUS

Nesreen Hamad[1, 2], Tsukasa Mashima[1, 2], Yudai Yamaoki[1], Keiko Kondo[1], Ryoma Yoneda[3], Takanori Oyoshi[4], Riki Kurokawa[3], Takashi Nagata[1, 2], and Masato Katahira[1, 2]*

[1] Institute of Advanced Energy, Kyoto University, Kyoto 611-0011, Japan

[2] Graduate School of Energy Science, Kyoto University, Kyoto 606-8501, Japan

[3] Research Center of Genomic Medicine, Saitama Medical University, Saitama 350-0495, Japan

[4] Department of Chemistry, Graduate School of Science, Shizuoka University, 836 Ohya, Suruga, Shizuoka 422–8529, Japan

* Corresponding author

E-mail: katahira@iae.kyoto-u.ac.jp

Supplementary Table S1. RNA/DNA sequences used in this study.

Name	Sequence
full length pncRNA	r(UUUUUCUAUCAGUUUUCUUUGAGCUUUUACUGUUAAGAGGGUACGG UGGUUUGAUGACACUGAACUAUAUUCAAAAGGAAGUAAAUGAACAGUU UUCUUAAUUUGGGGCAGGUACUGUAAAAAUAAAAACAAAAGUUAAGAC AGUAAAAUGUCCUUUUAUUUUUUAAUGCACCAAAGAGACAGAACCUGU AAUUUUAAAAACUGUGUAUUUUAAUUUACAUCUGCUUAAGUUUGCGAU AAUAUUGGGGACCCUCUCAUGUAACCACGAACACCUAUCGAUUUUGCU AAAAAUCAGAUCAGUACACUCGUUUGUUUAAUUGAUAAUUGUUCUGAA UUAUGCCGGCUCCUGCCAGCCCCCUCACGCUCACGAAUUCAGUCCCAG GGCAAAUUCUAAAGGUGAAGGGACGUCUACACCCCCAACAAAACCAAU UAGGAACCUUCGGUGGUCUUGUCCCAGGCAGAGGGGACUAAUAUUUCC AGCAAUUUAAUUUCUUUUUUAAUUAAAAAAAAUGAGUCAGAAUGGAGA UCACUGUUUCUCAGCUUUCCAUUCAGAGGUGUGUUUCUCCCGGUUAAA UUGCCGGCACGGGAAGGGAGGGGGUGCA)
pncRNA (R31)	r(GUUAAGAGGGUACGGUGGUUUGAUGACACUG)
5`-pncRNA (R13)	r(GUUAAGAGGGUAC)
3`-pncRNA (R19)	r(CGGUGGUUUGAUGACACUG)
R3	r(GGU)
R4	r(GGGU)
R5	r(AGGGU)
R6	r(GAGGGU)
R7	r(GUUAAGA)
R10	r(GGUUUGAUGA)
R12	r(GUUAAGAGGGUA)
U_{13}	r(UUUUUUUUUUUUU)
U_{19}	r(UUUUUUUUUUUUUUUUUUU)
U_{31}	r(UUUUUUUUUUUUUUUUUUUUUUUUUUUUU)
D31	d(GTTAAGAGGGTACGGTGGTTTGATGACACTG)
D13	d(GTTAAGAGGGTAC)
D19	d(CGGTGGTTTGATGACACTG)
TERRA	r(UUAGGGUUAGGGUUAGGGUUAGGG)
Mut_TERRA	r(UUAGGGUUAGUGUUAGUGUUAGGG)
Htelo	d(TTGGGTTAGGGTTAGGGTTAGGGA)
Mut_Htelo	d(TTGGGTTAGTGTTAGTGTTAGGGA)

Supplementary Table S2. Primer pairs used for PCR amplification (TagBFP and TagGFP2) and site directed mutagenesis (P525L and K510R).

TagBFP	forward	5′ CGCGGATCCGGTAGCGGCAGCGGTAGCATGAGCGAGCTGATTAAGGAGAAC 3′
	reverse	5′ CGCACCGGTAAGCTTGTGCCCCAGTTTGCTAG 3′
TagGFP2	forward	5′ CGGGGTACCATGAGCGGGGGCGAGGAG 3′
	reverse	5′ CTAGTCTAGACCTGTACAGCTCGTCCATGCCGTG 3′
P525L	forward	5′ TGTATGGTAGCGGCAGCGGTACCATGAGCG 3′
	reverse	5′ CGCTACCATACAGCCTCTCCCTGCGATCC 3′
K510R	forward	5′ GGATGGATTCCAGGGGTGAGCACAGACAGG 3′
	reverse	5′ GGAATCCATCCTGCCAGGGCCAAAGCCACC 3′

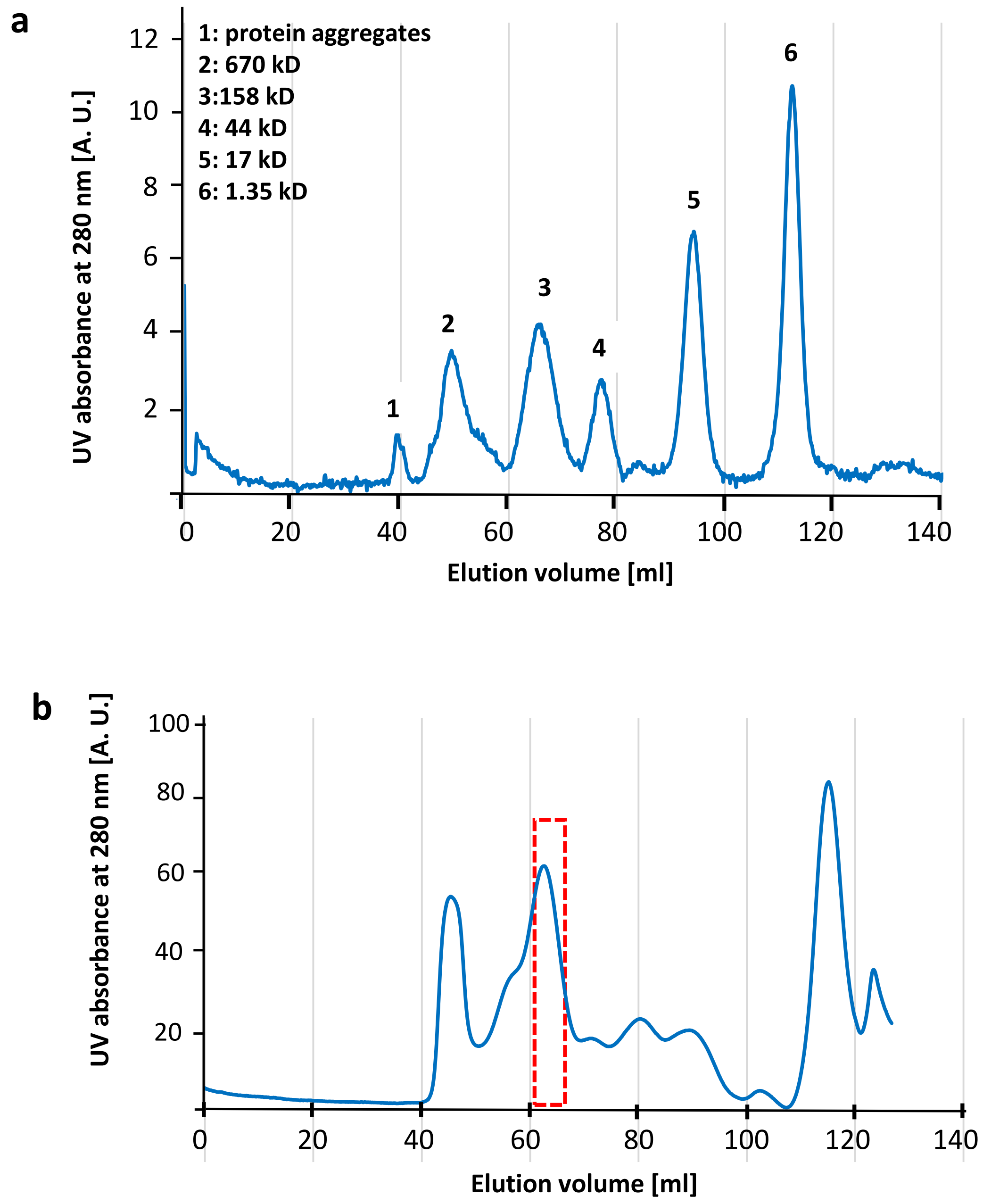

Supplementary Fig. S1: Purification of TLS as a monomer form.

a, Size-exclusion chromatography (SEC) of molecular size marker proteins. The theoretical molecular weigh of proteins is also indicated. **b**, SEC of TLS. Fractions corresponding to monomer TLS indicated with the red rectangular were collected.

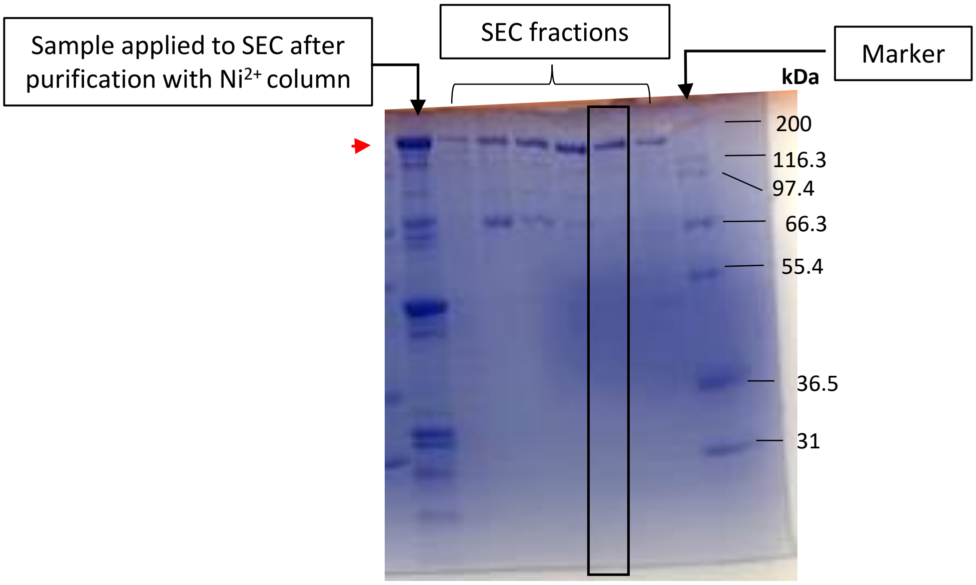

Supplementary Fig. S2: The uncropped full-length gel of 10% SDS-PAGE for fractions of MBP-BFP-TLS-GFP-6xHis eluted from size exclusion chromatography (SEC) and the molecular mass markers. The lane surrounded by a rectangular corresponds to the right lane of Fig. 1D. The fraction corresponding to this lane was used for FRET experiments. Red arrow indicates the position of MBP-BFP-TLS-GFP-6xHis.

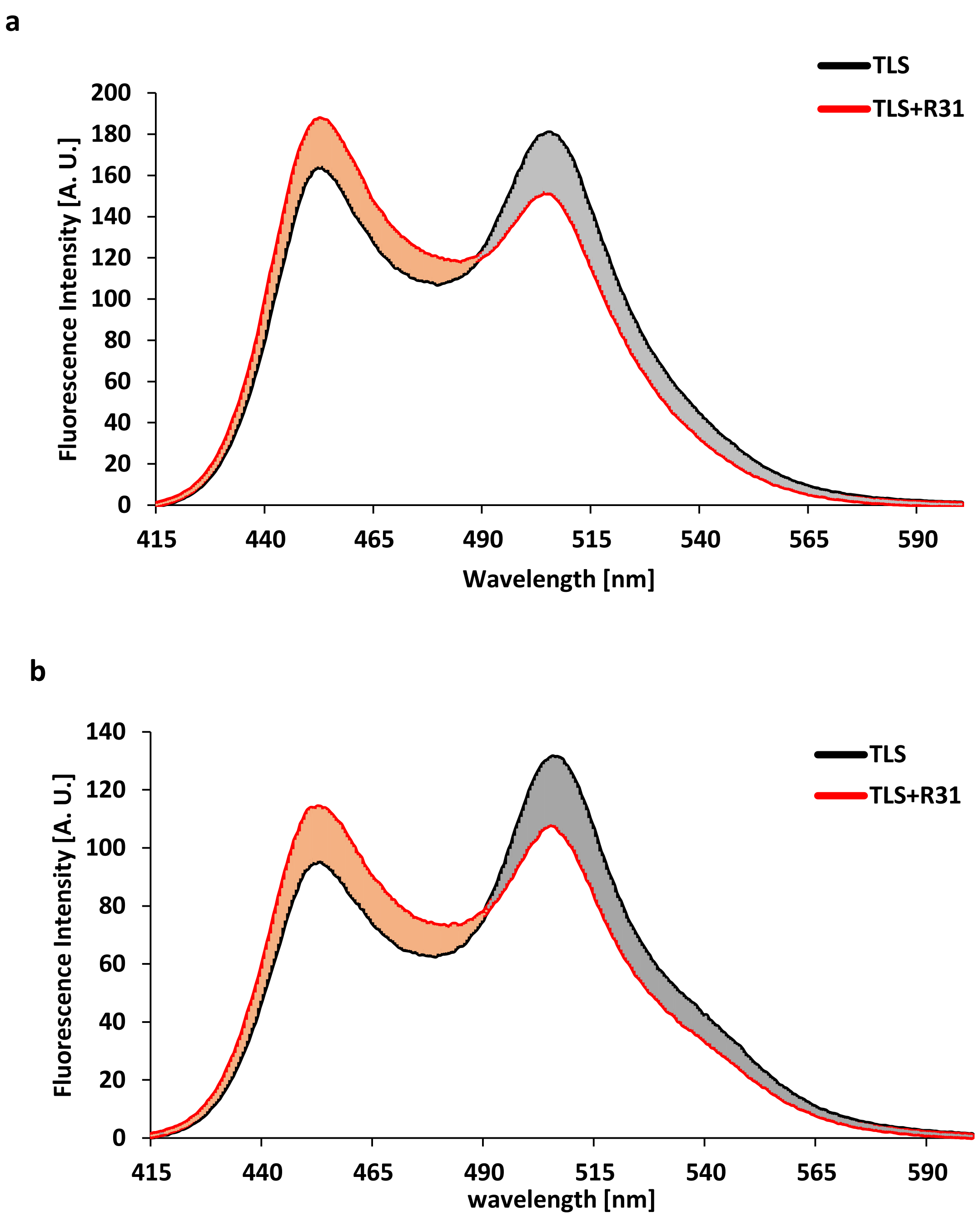

Supplementary Fig. S3: Little effect of the fusion of MBP on the FRET results of BFP-TLS-GFP. Fluorescence spectra of BFP-TLS-GFP with and without pncRNA (R31) in the presence (**a**) and absence (**b**) of the MBP fusion. The areas corresponding to the increase (orange) and decrease (gray) in fluorescence by the addition of R31 are colored.

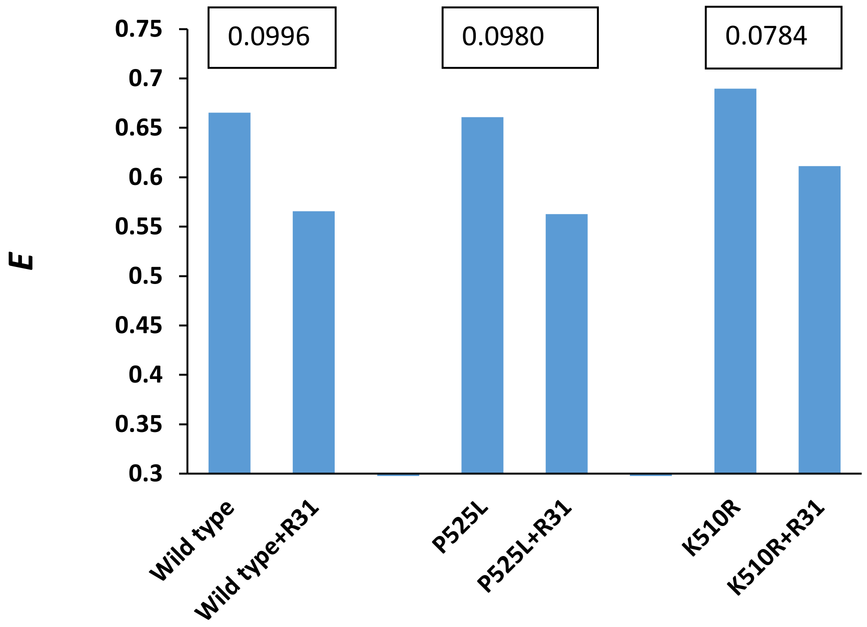

Supplementary Fig. S4: The conformation of TLS and its change by pncRNA (R31) are not affected by ALS-linked mutations of TLS. The *E* values in the absence and presence of R31 for wild-type and mutant TLSs are shown, each $|\Delta E|$ value being indicated in a box.
